# Hepatotoxicity of new-generation ALK inhibitors versus crizotinib in patients with non-small cell lung cancer: A systematic review and meta-analysis

**DOI:** 10.1016/j.isci.2025.114613

**Published:** 2026-01-02

**Authors:** Xingxian Luo, Xin Du, Qi Chen, Cen Wang, Lizong Li, Xu He, Yiru Gong, Jiali Chen, Xue Zhong, Yi Liu, Xiaohong Zhang, Lin Huang

**Affiliations:** 1 Department of Pharmacy, Peking University People’s Hospital, Beijing, China; 2 Vanke School of Public Health, Tsinghua University, Beijing, China; 3 School of Pharmaceutical Sciences, Tsinghua University, Beijing, China; 4 Liangzhu Laboratory, Zhejiang University, Hangzhou 310023, China

**Keywords:** Health sciences, Medicine, Medical specialty, Internal medicine, Oncology

## Abstract

Hepatotoxicity is a known side effect of ALK inhibitors in non-small cell lung cancer. This meta-analysis assessed the hepatotoxicity risk, measured by elevations in alanine aminotransferase (ALT) and aspartate aminotransferase (AST), for new-generation ALK inhibitors versus crizotinib through eight randomized controlled trials with 2,114 patients. The results suggested that new-generation ALK inhibitors were associated with a significantly reduced risk of all-grade ALT elevation (RR = 0.73, 95% confidence interval [CI] [0.58, 0.92]) and a trend toward reduced risk of grades 3–5 ALT elevation (RR = 0.55, 95% CI [0.29, 1.06]). Alectinib, lorlatinib, and brigatinib are associated with lower risks of hepatotoxicity when compared with crizotinib. New-generation ALK inhibitors improved progression-free survival but not in discontinuation rates. Lorlatinib conferred a greater reduction in any grades AST than second-generation inhibitors compared to crizotinib. Our findings suggest that the selection of the ALK inhibitor should be individualized based on the specific profile of hepatotoxicity and their efficacy.

## Highlights

• Alectinib, lorlatinib, and brigatinib have lower hepatotoxicity risk than crizotinib
• Envonalkib has higher risk of severe hepatotoxicity elevation than crizotinib
• Lorlatinib has lower hepatotoxicity risk and better PFS than 2^nd^-generation ALK inhibitors
• New ALK inhibitors have better PFS than crizotinib, but similar discontinuation rates

## Graphical abstract

**Figure:**
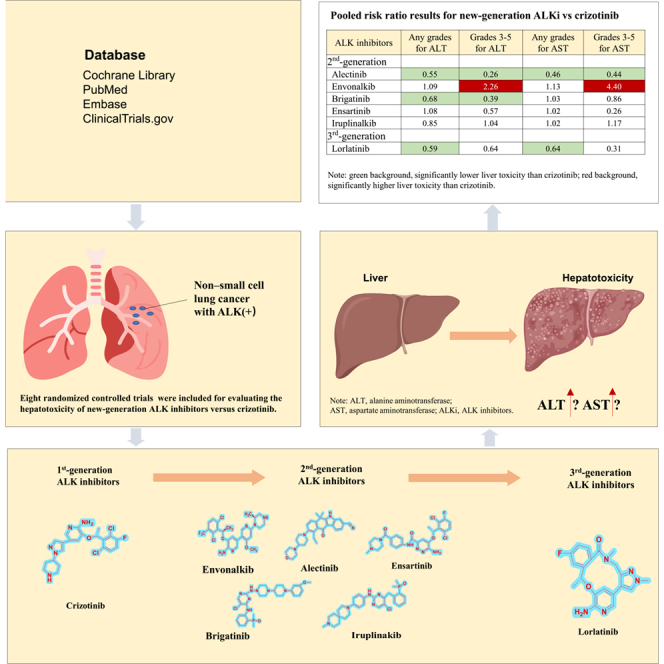

## Introduction

Anaplastic lymphoma kinase (ALK) inhibitors play a central role in the treatment of ALK-positive non-small cell lung cancer (NSCLC), a molecular subtype defined by ALK gene rearrangements.^1^^,^^2^ Crizotinib, the first-generation ALK tyrosine kinase inhibitor, demonstrated significant clinical activity but was limited by poor central nervous system (CNS) penetration and the rapid emergence of resistance.^1^^,^^3^ A principal mechanism is ALK-dependent resistance, characterized by the acquisition of secondary mutations within the ALK kinase domain (e.g., G1202R and L1196M), which impair drug binding and restore oncogenic signaling.^4^^,^^5^^,^^6^ To address these limitations, several second-generation (2^nd^) ALK inhibitors, such as alectinib,^7^ brigatinib,^8^ ensartinib,^9^ envonalkib,^10^ iruplinalkib,^9^^,^^11^ and third-generation (3^rd^) ALK inhibitors, such as lorlatinib^12^ have been developed and shown superior efficacy over crizotinib in randomized trials, with improved progression-free survival (PFS) and CNS activity. As of June 30, 2025, a total of eight ALK inhibitors have been approved globally, with six approved by the US Food and Drug Administration and all eight approved by China’s National Medical Products Administration (Table S1).

Like all targeted therapies, ALK inhibitors are associated with a spectrum of adverse events (AEs). Commonly reported toxicities include gastrointestinal disturbances (e.g., nausea, vomiting, and diarrhea), cardiovascular effects (e.g., bradycardia), and pulmonary toxicity (e.g., interstitial lung disease).^13^ Of particular concern is hepatotoxicity, specifically drug-induced liver injury (DILI), which has been frequently reported with tyrosine kinase inhibitors (TKIs), including ALK inhibitors.^14^^,^^15^^,^^16^ Crizotinib, the first-generation ALK inhibitor, is well known for its hepatotoxic effects, including elevations in liver transaminases and, in rare cases, severe DILI such as fulminant hepatic failure or even death.^13^^,^^17^ Clinical study has shown that up to 38% of patients treated with crizotinib experience liver-related AEs of any grade, with approximately 16% being Grade ≥ 3 AEs.^3^ In addition, our preliminary review of published case reports of severe crizotinib-induced liver injury revealed that over 40% (7/16) of the patients experienced fatal outcomes, highlighting the need for increased clinical vigilance regarding hepatotoxicity (Table S2).

Although new-generation ALK inhibitors have demonstrated significantly improved efficacy in treating ALK-positive NSCLC, hepatotoxicity remains a clinical concern. This risk is illustrated by documented serious hepatotoxicity cases involving 2^nd^-generation agents, such as alectinib, brigatinib, and ceritinib (Table S2).^13^ Also, regulatory authorities require routine liver function monitoring for all approved ALK inhibitors, as reflected in their prescribing information (Table S1). However, it remains unclear whether new-generation ALK inhibitors carry a lower, comparable, or even higher risk of hepatotoxicity compared to crizotinib. Therefore, a systematic comparative evaluation using meta-analysis is warranted to clarify the relative hepatotoxic risk among different ALK inhibitors, thereby guiding clinical decision-making and optimizing risk-benefit assessments.

The aim of this study was to evaluate the risk of all-grade and high-grade hepatotoxicity associated with six new-generation ALK inhibitors (2^nd^ ALK inhibitors: alectinib, brigatinib, ensartinib, iruplinalkib, envonalkib and 3^rd^ ALK inhibitors: lorlatinib) compared with the first-generation ALK inhibitor (crizotinib) in patients with ALK-positive NSCLC. Drug-specific risk estimates were also calculated through stratified analyses by individual ALK inhibitor. To provide a comprehensive assessment of the risk-benefit profile, PFS, response rate (RR), and discontinuation rate due to AEs were also analyzed.

## Results

### Search results

**Figure 1:**
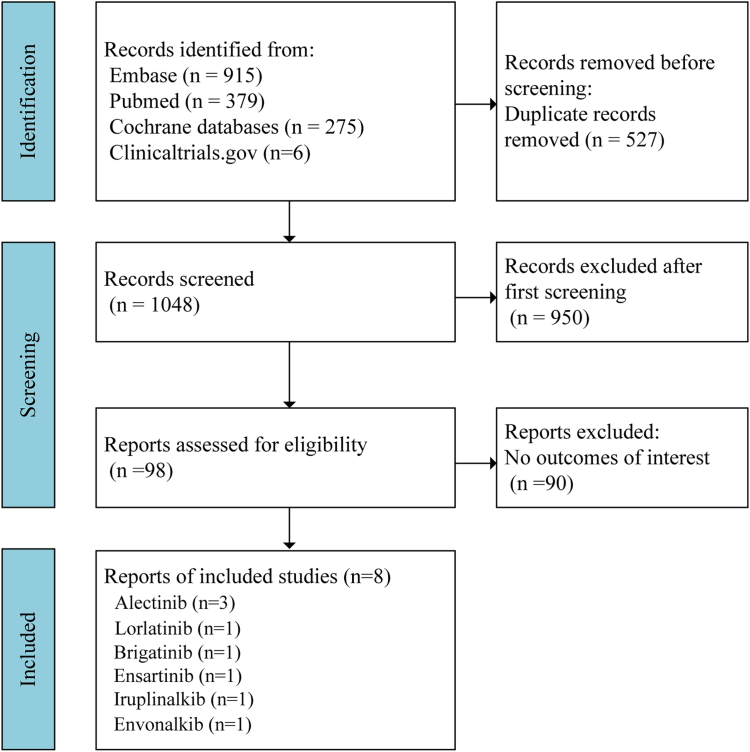
Flowchart for studies inclusion

A total of 1,575 records were identified through database searches, including Embase (*n* = 915), PubMed (*n* = 379), Cochrane databases (*n* = 275), and ClinicalTrials.gov (*n* = 6). After removal of 527 duplicate records, 1,048 records underwent title and abstract screening. Of these, 950 were excluded for irrelevance. The full texts of 98 reports were assessed for eligibility, of which 90 were excluded due to having no outcomes of interest. Finally, 8 studies were included in the analysis, comprising trials of alectinib (*n* = 3),^7^^,^^18^^,^^19^ lorlatinib (*n* = 1),^12^ brigatinib (*n* = 1),^8^ ensartinib (*n* = 1),^9^ irupinalkib (*n* = 1),^11^ and envonalkib (*n* = 1)^10^ (Figure 1).

### Characteristics of the studies and quality assessment

Table 1 summarizes the characteristics of the included clinical trials and their quality assessment results. In total, 8 RCTs comparing ALK inhibitors with crizotinib in treatment-naive ALK-positive NSCLC were included, comprising five multi-regional clinical trials,^7^^,^^8^^,^^9^^,^^12^^,^^19^ two conducted exclusively in China,^11^ and one exclusively in Japan.^10^ Trial enrollment ranged from 187 to 303 patients, and crossover between study arms was allowed in 2 studies.^8^^,^^18^ The primary efficacy endpoint in all trials was PFS. The methodological quality of each study was assessed using the Jadad scale, which evaluates randomization, blinding, and dropout reporting (range, 0–5). Among the 8 trials, 4 studies were rated as high quality with a Jadad score of 5 (CROWN,^12^ ALTA-1L,^8^ INSPIRE,^11^ and the TQ-B3139^10^ trial), owing to the use of appropriate randomization procedures and blinded independent central review of efficacy outcomes. The remaining 4 studies (ALEX,^7^ J-ALEX,^18^ ALESIA,^19^ and eXalt3^9^) received a score of 3 due to open-label design without blinded assessment. All trials reported patient withdrawals or dropouts. No evidence of publication bias was detected for primary outcomes, as indicated by funnel plots and the Egger test (Figure S1). The results of the sensitivity analysis showed that the pooled estimates for ALT and AST remained stable after the removal of each individual study (data not shown).

**Table 1:** Characteristic of studies included in the meta-analysis

Source	Trial number	Trial name	Treatment group	Control group	Trial design	Primary efficacy endpoints	Crossover between study groups	Enrolled patients	Females, no./males, no.	Jadad score
Peters et al.^7^	NCT02075840	ALEX	Alectinib, 600 mg BID	Crizotinib, 250 mg BID	MRCT	PFS	Not allowed	303	164/139	3
Hida et al.^18^	JapicCTI-132316	J-ALEX	Alectinib, 600 mg BID	Crizotinib, 250 mg BID	Only in Japan	PFS	Allowed	207	82/125	3
Zhou et al.^19^	NCT02838420	ALESIA	Alectinib, 300 mg BID	Crizotinib, 250 mg BID	MRCT	PFS	Not allowed	187	98/89	3
Shaw et al.^12^	NCT03052608	CROWN	Lorlatinib, 100 mg QD	Crizotinib, 250 mg BID	MRCT	PFS	Not allowed	296	175/121	5
Horn et al.^9^	NCT02767804	eXalt3	Ensartinib, 225 mg QD	Crizotinib, 250 mg BID	MRCT	PFS	Not allowed	290	149/161	3
Camidge et al.^8^	NCT02737501	ALTA-1L	Brigatinib, 180 mg QD	Crizotinib, 250 mg BID	MRCT	PFS	Allowed	275	150/125	5
Shi et al.^11^	NCT04632758	INSPIRE	Iruplinalkib, 180 mg QD	Crizotinib, 250 mg BID	Only in China	PFS	Not allowed	292	134/158	5
Yang et al.^10^	NCT04009317	TQ-B3139	Envonalkib, 600 mg BID	Crizotinib, 250 mg BID	Only in China	PFS	NR	264	136/128	5

MRCT, multiregional clinical trials; PFS, progression-free survival; NR, not reported; BID, twice a day; QD, once a day.

### Primary outcomes

Table 2 summarizes the pooled RR results for the primary outcomes, including any-grade and grades 3–5 AEs for ALT and AST elevation.

**Table 2:** RR of hepatotoxicity associated with ALK inhibitors in the treatment of ALK-positive NSCLC

ALK inhibitors	RR (95% CI)	I^2^ statistic, %	*p* value
**Any grades of ALT elevation**			

2^nd^-generation ALK inhibitors			
Alectinib	0.55 (0.44, 0.70)	80	**0.011**
Envonalkib	1.09 (0.94, 1.27)	NA	0.251
Brigatinib	0.68 (0.57, 0.82)	NA	**<0.001**
Ensartinib	1.08 (0.85, 1.39)	NA	0.525
Iruplinalkib	0.85 (0.69, 1.05)	NA	0.138
3^rd^-generation ALK inhibitors			
Lorlatinib	0.59 (0.48, 0.72)	NA	**<0.001**
Overall	0.73 (0.58, 0.92)	85	0.007

**Grades 3–5 of ALT elevation**			

2^nd^-generation ALK inhibitors			
Alectinib	0.26 (0.13, 0.51)	0	**<0.001**
Envonalkib	2.26 (1.07, 4.77)	NA	0.033
Brigatinib	0.39 (0.17, 0.91)	NA	**0.029**
Ensartinib	0.57 (0.19, 1.65)	NA	0.298
Iruplinalkib	1.04 (0.48, 2.24)	NA	0.916
3^rd^-generation ALK inhibitors			
Lorlatinib	0.64 (0.18, 2.20)	NA	0.475
Overall	0.55 (0.29, 1.06)	68.2	0.073

**Any grades of AST elevation**			

2^nd^-generation ALK inhibitors			
Alectinib	0.46 (0.29, 0.74)	31	**0.001**
Envonalkib	1.13 (0.95, 1.34)	NA	0.183
Brigatinib	1.03 (0.88, 1.20)	NA	0.718
Ensartinib	1.02 (0.76, 1.40)	NA	0.892
Iruplinalkib	1.02 (0.84, 1.23)	NA	0.851
3^rd^-generation ALK inhibitors			
Lorlatinib	0.64 (0.53, 0.78)	NA	**<0.001**
Overall	0.83 (0.67, 1.04)	83	0.112

**Grades 3–5 of AST elevation**			

2^nd^-generation ALK inhibitors			
Alectinib	0.44 (0.21, 0.95)	0	**0.036**
Envonalkib	4.40 (1.28, 15.08)	NA	**0.018**
Brigatinib	0.86 (0.30, 2.50)	NA	0.787
Ensartinib	0.26 (0.03, 2.26)	NA	0.219
Iruplinalkib	1.17 (0.47, 2.95)	NA	0.736
3^rd^-generation ALK inhibitors			
Lorlatinib	0.31 (0.09, 1.12)	NA	0.074
Overall	0.81 (0.41, 1.59)	51	0.541

RR, risk ratio; ALT, alanine aminotransferase; AST, aspartate aminotransferase; ALK, anaplastic lymphoma kinase; NSCLC, non-small cell lung cancer; CI, confidence interval. Bold font indicates a *p* value of less than 0.05.

### ALT elevation

**Figure 2:**
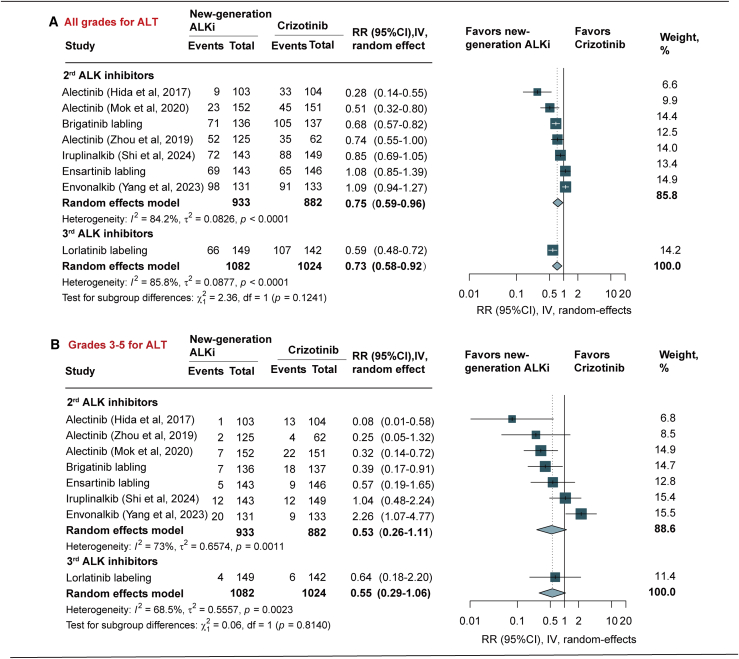
Pooled RR of all-grade and grades 3–5 alanine aminotransferase elevation of crizotinib versus new ALK inhibitors IV, inverse variance method; RR, risk ratio; ALKi, anaplastic lymphoma kinase inhibitors. Data are represented as RR and 95% CI.

Among patients treated with new-generation ALK inhibitors, the risk of all-grade ALT elevation was significantly lower compared with crizotinib (RR = 0.73, 95% CI [0.58, 0.92], *p* = 0.007, *I*^2^ = 85.8%; Figure 2A). Subgroup analysis for 2^nd^-generation ALK inhibitors showed that alectinib (RR = 0.55, 95% CI [0.44, 0.70], *p* < 0.011) and brigatinib (RR = 0.68, 95% CI [0.57, 0.82], *p* < 0.001) were associated with significantly lower incidence of ALT elevation. In contrast, ensartinib (RR = 1.08, 95% CI [0.85, 1.39], *p* = 0.525), iruplinalkib (RR = 0.85, 95% CI [0.69, 1.05], *p* = 0.138), and envonalkib (RR = 1.09, 95% CI [0.94, 1.27], *p* = 0.251) did not show statistically significant differences compared with crizotinib. For 3^rd^-generation ALK inhibitors, lorlatinib significantly reduced the risk of ALT elevation (RR = 0.59, 95% CI [0.48, 0.77], *p* < 0.001) (Table 2).

For grades 3–5 ALT elevation, the new-generation ALK inhibitors demonstrated a non-significant trend toward reduced risk (RR = 0.55, 95% CI [0.29, 1.06], *p* = 0.073, *I*^2^ = 68.5%). Subgroup analysis for 2^nd^-generation ALK inhibitors showed that alectinib (RR = 0.26, 95% CI [0.13, 0.51], *p* < 0.001) and brigatinib (RR = 0.39, 95% CI [0.17, 0.91], *p* = 0.029) significantly reduced the risk of grades 3–5 ALT elevation. Conversely, envonalkib was associated with a significantly increased risk (RR = 2.26, 95% CI [1.07, 4.77], *p* = 0.033), whereas ensartinib and iruplinalkib showed no statistically significant difference from crizotinib. For 3^rd^-generation ALK inhibitor, lorlatinib did not significantly reduce the risk of grades 3–5 ALT elevation (RR = 0.64, 95% CI [0.18, 2.20], *p* = 0.475) (Figures 2B; Table 2).

Compared to crizotinib, both 2^nd^- and 3^rd^-generation ALK inhibitors are similar to each other in reducing the risk of any grade (*p* = 0.124) and grades 3–5 ALT elevation (*p* = 0.814) (Figures 2A and 2B).

### AST elevation

**Figure 3:**
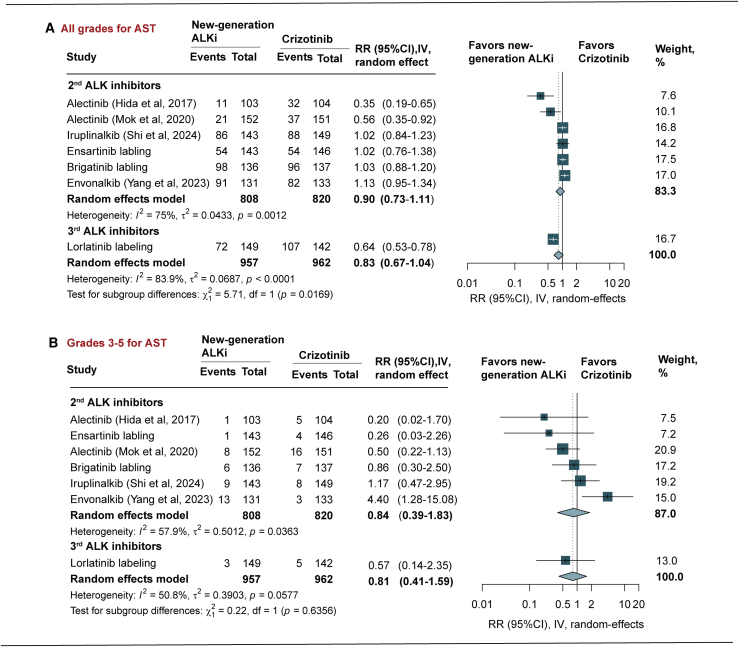
Pooled RR of all-grade and grades 3–5 aspartate aminotransferase elevation IV, inverse variance method; RR, risk ratio; ALKi, anaplastic lymphoma kinase inhibitors. Data are represented as RR and 95% CI.

The risk of all-grade AST elevation with new-generation ALK inhibitors compared with crizotinib was not significant (RR = 0.83, 95% CI [0.67, 1.04], *p* = 0.112, *I*^2^ = 83.9%). For 2^nd^-generation ALK inhibitors, alectinib showed a significant reduction (RR = 0.46, 95% CI [0.29, 0.74], *p* = 0.001) while brigatinib (RR = 1.03, 95% CI [0.88, 1.20], *p* = 0.718), ensartinib (RR = 1.02, 95% CI [0.76, 1.40], *p* = 0.892), iruplinalkib (RR = 1.02, 95% CI [0.84, 1.23], *p* = 0.851), and envonalkib (RR = 1.13, 95% CI [0.95, 1.34], *p* = 0.183) showed no significant differences compared to crizotinib. For 3^rd^-generation ALK inhibitor, lorlatinib significantly reduced the risk of AST elevation (RR = 0.64, 95% CI [0.53, 0.78], *p* < 0.001) (Figure 3A; Table 2).

For grades 3–5 AST elevation, the new-generation ALK inhibitors were not associated with a significant reduction in risk compared to crizotinib (RR = 0.81, 95% CI [0.41, 1.59], *p* = 0.392, *I*^2^ = 50.8%). Subgroup analysis for 2^nd^-generation ALK inhibitors showed that alectinib (RR = 0.44, 95% CI [0.21, 0.95], *p* = 0.036) and significantly reduced the risk of severe AST elevation. In contrast, envonalkib was associated with a significantly increased risk of grades 3–5 AST elevation (RR, 4.40, 95% CI [1.28, 15.08], *p* = 0.018), whereas brigatinib, ensartinib, and iruplinalkib did not significantly differ from crizotinib. For 3^rd^-generation ALK inhibitor, lorlatinib showed a non-significant benefit (RR = 0.31, 95% CI [0.09, 1.12], *p* = 0.074) (Figure 3B; Table 2).

Compared to crizotinib, 3^rd^-generation ALK inhibitors were associated with an even greater reduction in any-grade AST than 2^nd^-generation inhibitors (*p* = 0.017), though no significant difference was observed for grades 3–5 AST (*p* = 0.636) (Figures 3A and 3B).

### Secondary outcomes

**Figure 4:**
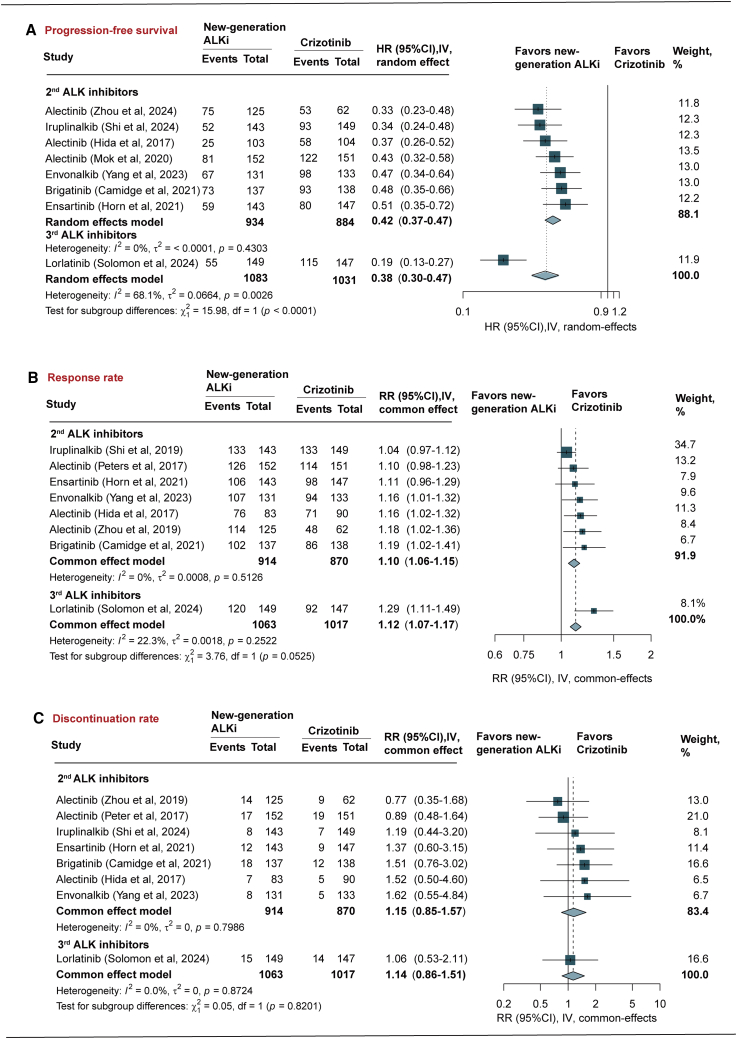
Pooled analysis of PFS, response rate, and discontinuation rate IV, inverse variance method; HR, hazard ratio; RR, risk ratio; ALKi, anaplastic lymphoma kinase inhibitors. Data are represented as HR or RR and 95% CI.

Table 3 and Figure 4 presents the pooled results of hazard ratio (HR) for PFS, risk ratio (RR) for objective response rate (ORR), and discontinuation rate for secondary outcomes.

**Table 3:** Pooled results of PFS, response rate, and discontinuation rate associated with ALK inhibitors in the treatment of NSCLC

ALK inhibitors	HR/RR (95% CI)	I^2^ statistic, %	*p* value
**Progression-free survival**			

2^nd^-generation ALK inhibitors			
Alectinib	0.39 (0.32, 0.47)	0	<0.001
Brigatinib	0.49 (0.35, 0.66)	NA	<0.001
Ensartinib	0.52 (0.36, 0.73)	NA	<0.001
Iruplinalkib	0.35 (0.24, 0.48)	NA	<0.001
Envonalkib	0.47 (0.34, 0.64)	NA	<0.001
3^rd^-generation ALK inhibitors			
Lorlatinib	0.20 (0.13, 0.27)	NA	<0.001
Overall	0.38 (0.31, 0.47)	68	<0.001

**Response rate**			

2^nd^-generation ALK inhibitors			
Alectinib	1.09 (1.03, 1.15)	16.8	0.001
Brigatinib	1.19 (1.02, 1.41)	NA	0.032
Ensartinib	1.11 (0.96, 1.29)	NA	0.165
Iruplinalkib	1.04 (0.97, 1.12)	NA	0.260
Envonalkib	1.16 (1.01, 1.32)	NA	0.037
3^rd^-generation ALK inhibitors			
Lorlatinib	1.29 (1.11, 1.49)	NA	0.001
Overall	1.12 (1.07, 1.17)	22.3	<0.001

**Discontinuation rate**			

2^nd^-generation ALK inhibitors			
Alectinib	0.69 (0.45, 1.05)	0	0.084
Envonalkib	1.62 (0.55, 4.84)	NA	0.384
Brigatinib	1.51 (0.76, 3.02)	NA	0.242
Ensartinib	1.37 (0.60, 3.15)	NA	0.458
Iruplinalkib	1.19 (0.44, 3.20)	NA	0.729
3^rd^-generation ALK inhibitors			
Lorlatinib	1.06 (0.53, 2.11)	NA	0.875
Overall	1.14 (0.86, 1.51)	0	0.370

ALK, anaplastic lymphoma kinase; PFS, progression-free survival; HR, hazard ratio; RR, risk ratio; ; CI, confidence interval; NA, not available.

### Progression-free survival

In pooled analysis of randomized trials, the new-generation ALK inhibitors were associated with a significantly prolonged PFS compared with crizotinib (HR = 0.38, 95% CI [0.31, 0.47], *p* < 0.001, *I*^2^ = 68.0%). Subgroup analyses of 2^nd^- and 3^rd^-generation ALK inhibitors demonstrated consistent and significant benefits. Lorlatinib exhibited the greatest reduction in risk of progression or death (HR = 0.19, 95% CI [0.13, 0.27], *p* < 0.001), followed by iruplinalkib (HR = 0.34, 95% CI [0.24, 0.48], *p* < 0.001), alectinib (HR = 0.39, 95% CI [0.32, 0.47], *p* < 0.001), envonalkib (HR = 0.47, 95% CI [ 0.34, 0.64], *p* < 0.001), brigatinib (HR = 0.49, 95% CI [0.35, 0.66], *p* < 0.001), and ensartinib (HR = 0.52, 95% CI [0.36, 0.73], *p* < 0.001). Lorlatinib was associated with a significantly improved PFS compared to 2^nd^-generation ALK inhibitors (*p* < 0.001) (Figure 4A; Table 3).

### Response rate

In pooled analysis, the new-generation ALK inhibitors were associated with a significantly higher of achieving ORR compared with crizotinib (RR = 1.12, 95% CI [1.07, 1.17], *p* < 0.001, *I*^2^ = 22.3%). Among individual agents, lorlatinib showed the superior in ORR rate (RR = 1.29, 95% CI [1.11, 1.49], *p* = 0.001), followed by brigatinib (RR = 1.19, 95% CI [1.02, 1.41], *p* = 0.032), alectinib (RR = 1.09, 95% CI [1.03, 1.15], *p* = 0.001), and envonalkib (RR = 1.16, 95% CI [1.01, 1.32], *p* = 0.037). Ensartinib (RR = 1.11, 95% CI [0.96, 1.29], *p* = 0.165) and iruplinalkib (RR = 1.04, 95% CI [0.97, 1.12], *p* = 0.260) did not demonstrate a statistically significant difference in ORR compared to crizotinib. The 2^nd^- and 3^rd^-generation ALK inhibitors showed comparable ORR when compared with the crizotinib (*p* = 0.053) (Figure 4B; Table 3).

### Discontinuation rate due to AEs

There was no statistically significant difference in the risk of discontinuation rate due to AEs between the new-generation ALK inhibitors and crizotinib (RR = 1.14, 95% CI [0.86, 1.51], *p* = 0.370, *I*^2^ = 0%). Specifically, among the 2^nd^-generation ALK inhibitors, the RR were 0.69 (95% CI [0.45, 1.05], *p* = 0.084) for alectinib, 1.62 (95% CI [0.55, 4.84], *p* = 0.384) for envonalkib, 1.51 (95% CI [0.76, 3.02], *p* = 0.242) for brigatinib, 1.37 (95% CI [0.60, 3.15], *p* = 0.458) for ensartinib, and 1.19 (95% CI [0.44, 3.20], *p* = 0.729) for iruplinalkib. For the 3^rd^-generation inhibitor lorlatinib, the RR was 1.06 (95% CI [0.53, 2.11], *p* = 0.875) (Figure 4C; Table 3).

## Discussion

TKIs are widely used in the treatment of various malignancies and are known to cause hepatotoxicity to varying degrees.^20^^,^^21^^,^^22^^,^^23^ A previous meta-analysis that included 12 studies reported that TKIs were associated with a significantly increased risk of high-grade hepatotoxicity compared with control treatments (OR = 4.35).^22^ Another meta-analysis involving 3,691 patients found that the incidence of hepatotoxicity related to anti-angiogenic targeted agents ranged from 23% to 40%, with 5% being classified as high-grade.^20^ Similarly, treatment with vascular endothelial growth factor receptor (VEGFR) inhibitors was associated with significantly higher risks of elevated ALT (RR = 1.57), AST (RR = 1.57), alkaline phosphatase (RR = 1.20), and bilirubin (RR = 1.55) levels compared with controls.^21^ Notably, a recent meta-analysis comparing first-generation and new-generation BCR-ABL inhibitors showed that new-generation inhibitors were associated with a higher risk of both any-grade ALT elevation (RR, 2.89) and high-grade ALT elevation (RR, 1.59), despite their improved response rates.^15^

Previous study summarized data from two ALK inhibitors in a pooled analysis of 1,908 patients, reporting that ALK TKIs were associated with elevations in ALT and AST of any grade in 25.2% and 26.0% of patients, respectively, and high-grade elevations in 7.0% and 9.9%.^23^ However, at that time, only two ALK inhibitors were approved, and all control groups received chemotherapy, limiting the ability to assess the relative hepatotoxicity of new-generation ALK inhibitors compared to first-generation crizotinib. This meta-analysis aimed to evaluate the risk of hepatotoxicity associated with ALK tyrosine kinase inhibitors in patients with ALK-positive NSCLC, with a direct comparison to crizotinib. The results showed that new-generation ALK inhibitors significantly reduced the risk of ALT elevation, while having no significant effect on AST levels. It is important to note that hepatotoxicity risk varies across different new-generation ALK inhibitors. Compared with crizotinib, alectinib, lorlatinib, and brigatinib were associated with a significantly lower risk of hepatotoxicity, whereas envonalkib was associated with an increased risk. These findings provide important evidence for selecting appropriate ALK inhibitors, particularly in patients who discontinued crizotinib due to hepatotoxicity.

Early identification of risk factors for ALK inhibitor-induced hepatotoxicity is crucial for guiding safe and effective treatment decisions. Several published studies, summarized in Table S4, have investigated factors associated with hepatotoxicity, primarily focusing on crizotinib. Jung et al. reported that crizotinib-induced hepatotoxicity may be associated with preexisting liver disease, hepatitis B virus infection, and concomitant use of H2-receptor antagonists or proton pump inhibitors (PPIs).^24^ Similarly, another study found that sex, liver metastases, and the use of H2 blockers or PPIs were potential risk factors for high-grade hepatoxiciy.^25^ Lin et al. reported that combining crizotinib with immune checkpoint inhibitors significantly increased the risk of hepatotoxicity, suggesting the need for close monitoring when crizotinib is used following immunotherapy.^26^ In addition, genetic susceptibility, such as the STAT1 rs10208033 polymorphism, has also been implicated in crizotinib-related liver injury.^27^ However, all current evidence is based on retrospective observational studies, highlighting the need for high-quality prospective studies to validate these findings.

The mechanisms underlying ALK inhibitors-induced hepatotoxicity was not fully understood but may involve multiple pathways (Table S4). Crizotinib has been shown to cause reactive oxygen species (ROS) accumulation and inhibit the Nrf2 antioxidant pathway, leading to mitochondrial dysfunction,^28^^,^^29^ pyroptosis via NF-κB/NLRP3,^30^ and ferroptosis through Stat1-mediated Nrf2 suppression.^31^ It also disrupts autophagosome-lysosome fusion and alters cholesterol/sphingolipid metabolism while blocking autophagic squalene epoxidase degradation further exacerbates metabolic disturbance and apoptosis.^32^ Earlier studies also reported non-mitochondrial apoptosis^32^ and ROS-mediated DNA damage independent of ALK targets.^33^ In additional, ensartinib induces liver injury mainly via TXNIP-mediated oxidative stress and mitochondrial dysfunction.^34^ These findings highlight the multifaceted mechanisms of ALK inhibitor-induced liver injury and emphasize the importance of identifying intervention strategies as a key focus for future research.

In recent years, the toxic side effects (including hepatotoxicity) caused by excessively high doses of targeted cancer drugs have attracted widespread attention.^35^^,^^36^ Traditionally, the dose selection of chemotherapy drugs relied on the maximum tolerated dose (MTD) or maximum administered dose (MAD), due to their steep dose-response curve. However, compared to chemotherapy drugs, targeted anticancer agents exhibit a shallower dose-response curve, suggesting that selecting the MTD may not be optimal.^37^^,^^38^ Additionally, since targeted therapies are administered over prolonged periods, excessively high doses may significantly impair the patients’ adherence and quality of life.^39^ To address this, the US FDA has established a dose optimization initiative (Project Optimus), encouraging the conduct of dose optimization studies, particularly RCT comparing different doses, before the approval of targeted anticancer drugs.^40^ Among the seven ALK inhibitors included in this study, two were approved at the MTD or MAD, while the rest were approved at doses lower than the MTD or MAD. Furthermore, only one of these agents underwent dose optimization through an RCT (eTable 5).^41^ Collectively, the evidence advocates for dose-optimization studies to improve the hepatic safety profile of ALK inhibitors.

### Limitations of the study

Several limitations should be considered when interpreting the findings of this meta-analysis. First, the analysis was based on aggregated study-level data rather than individual patient data, which precluded detailed evaluation of patient-specific risk modifiers, such as baseline liver function status, genetic polymorphisms, or prior hepatic disease. Second, although transaminase elevation was the primary indicator of hepatotoxicity, other relevant hepatic biomarkers and clinical outcomes, such as jaundice, bilirubin elevation, and liver failure events, were not evaluated due to limited data. Third, this study only evaluated efficacy outcomes of ORR and PFS, but did not include OS primarily because OS data from most studies were still immature. Therefore, OS outcomes should be incorporated into future risk-benefit assessments. Lastly, data from real-world settings and non-English publications were not included, which may limit the generalizability of the results to broader clinical populations.

This meta-analysis highlights the variability in hepatotoxicity risk among the new-generation ALK inhibitors. Alectinib, lorlatinib, and brigatinib are associated with lower risks of hepatotoxicity when compared with crizotinib. However, envonalkib shows a significantly increased risk of grades 3–5 ALT and AST elevation compared with crizotinib. These findings suggest that new-generation ALK inhibitors do not uniformly reduce hepatotoxicity. Additionally, the cases of hepatotoxicity induced by ALK inhibitors with fatal outcomes primarily associated with crizotinib, suggest that close monitoring of its use is warranted. The mechanisms underlying ALK inhibitor-induced hepatotoxicity still require further elucidation. However, these findings are constrained by the limited number of studies and small sample sizes. Future large-scale, multi-center RCTs with extended follow-up are required to validate these results.

## Resource availability

### Lead contact

Further information and requests for resources and reagents should be directed to and will be fulfilled by the lead contact, Lin Huang (huanglin@pkuph.edu.cn).

### Materials availability

This study did not generate new unique reagents.

### Data and code availability

• This study did not generate any new original datasets that require public deposition. The data that support the findings of this study are available from the corresponding author upon reasonable request.
• No custom code was generated; all analyses were performed using publicly available software as detailed in the key resources table.
• No additional unique resources requiring specialized access procedures were generated in this study.

## Acknowledgments

This work was supported by the 10.13039/501100001809National Natural Science Foundation of China (ref. 72304168) and Peking University People’s Hospital Development Foundation (RS2024-05).

## Author contributions

X.L., X.D., and L.H. conceptualized and designed the study; X.L., X.D., and Q.C. conducted the literature review and performed the statistical analysis; C.W., L.L., X.H., Y.G., and J.C. collected the data; X.L. and X.D. drafted the manuscript; X.Z., Y.L., and L.H. reviewed and edited the manuscript; X.H.Z. and L.H. supervised the project.

## Declaration of interests

The authors declare no competing interests.

## STAR★Methods

### Key resources table

**Table:** 

REAGENT or RESOURCE	SOURCE	IDENTIFIER
**Deposited data**		

Cochrane Library	Cochrane Library	https://www.cochranelibrary.com/
PubMed	PubMed	https://pubmed.ncbi.nlm.nih.gov/
Embase	Embase	https://www.embase.com/
ClinicalTrials.gov	ClinicalTrials.gov	https://www.clinicaltrials.gov/

### Experimental model and study participant details

The study did not use experimental models typical in the life sciences.

### Method details

#### Definition of the outcomes

Hepatotoxicity served as the primary outcomes, with comprehensive assessment through serial monitoring of serum hepatic transaminase levels. Specifically, we analyzed both all-grade elevations and clinically significant Grade ≥3 elevations in alanine aminotransferase (ALT) and aspartate aminotransferase (AST), as defined by the Common Terminology Criteria for Adverse Events (CTCAE) guidelines. For this analysis, the term “new-generation ALK inhibitors” encompassed both 2^nd^-generation agents (alectinib, brigatinib, ensartinib, iruplinalkib and envonalkib) and 3^rd^-generation agents (lorlatinib). The hepatotoxicity of new-generation ALK inhibitors was systematically compared with that of crizotinib. Additionally, data on ORR, PFS and discontinuation rate of ALK inhibitors therapy due to adverse events were taken as the secondary outcomes.

#### Search strategy and study selection

A comprehensive search was conducted across PubMed, Embase, Cochrane Library and ClinicalTrials.gov from the inception to June 1, 2025. The search strategy incorporated specific keywords and MeSH terms related to “ALK inhibitors” and “non-small cell lung cancer” (NSCLC), with full search syntax and results for each database provided in supplement (Appendix S1; Table S3). Only English-language publications considered (Appendix S2; Table S3 in the supplement). The search was supplemented by reviewing completed trials listed on ClinicalTrials.gov up to June 2025. This study followed the Preferred Reporting Items for Systematic Reviews and Meta-analyses (PRISMA) guidelines.^42^ The predefined inclusion and exclusion criteria were registered in the International Prospective Register of Systematic Reviews (PROSPERO: CRD420251079727). In cases where trials had multiple publications, we selected only the most recent and comprehensive report for inclusion. Study quality was assessed using the Jadad score.^43^ This systematic review, utilizing only publicly available published data, did not require IRB approval or patient consent (Figure 1).

#### Data extraction

Two investigators independently conducted data extraction with consensus resolution of discrepancies. The extraction process captured detailed study characteristics including authorship, publication year, trial designs, sample demographics, regimens, dosing schedules, administration frequencies, ALT levels, AST levels, RR, PFS and discontinuation rate related to the AEs. For any missing data in the published literature, we referred to the most up-to-date official drug prescribing information (package insert) as a supplementary source.

### Quantification and statistical analysis

Risk ratio (RR) and hazard ratio (HR) with corresponding 95% confidence intervals (CIs) were calculated using the inverse variance method. For studies where the outcomes were not reported with a 95% confidence interval (95% CI), we converted the estimates to 95% CI for consistency (Appendix S3 in the supplement). Heterogeneity among studies was assessed using the Q statistic and *I*^*2*^ statistic. When significant heterogeneity was detected (*p* < 0.10 for the Q-test or *I*^*2*^ > 50%), a random-effects model was employed; otherwise, a fixed-effects model was used. Publication bias was evaluated through funnel plots and Egger’s regression test. Furthermore, we conducted a pooled analysis for each ALK inhibitor separately. Sensitivity analysis was performed by sequentially removing each individual study to evaluate the stability of the overall results. A two-sided *p*-value <0.05 was considered statistically significant. All statistical analyses were conducted using R, version 4.1.0 (R Project for Statistical Computing) with the meta and metafor packages. All analyses were performed between June 2025 and July 2025.

## Supplemental information

Document S1. Figure S1, Tables S1–S5, and Appendices S1–S3
