# Supplementary material for: Hepatotoxicity of new-generation ALK inhibitors versus crizotinib in patients with non-small cell lung cancer: A systematic review and meta-analysis
Source: iScience. 2026 Jan 2;29(2):114613. doi: 10.1016/j.isci.2025.114613 (PMC12855586; doi:10.1016/j.isci.2025.114613)
Supplement: Document S1. Figure S1, Tables S1–S5, and Appendices S1–S3 [file mmc1.pdf]

iScience, Volume *29*

Supplemental information

Hepatotoxicity of new-generation ALK inhibitors versus crizotinib in patients with non-small cell lung cancer: A systematic review and meta-analysis

Xingxian Luo (罗兴献), Xin Du (杜鑫), Qi Chen (陈琪), Cen Wang (王岑), Lizong Li (李理总), Xu He (何旭), Yiru Gong (龚怡如), Jiali Chen (陈佳丽), Xue Zhong (钟雪), Yi Liu (刘一), Xiaohong Zhang (张晓红), and Lin Huang (黄琳)

SUPPLEMENTARY MATERIALS

Appendix S1. Evidence sources and search strategy.

A systematic search adhering to PICO criteria was performed in Embase, PubMed, the Cochrane Library, and ClinicalTrials.gov from inception until June 1, 2025. XXL and XD independently screened records by title, abstract, and full text against the inclusion criteria. Discrepancies were resolved through adjudication by a third reviewer.

Participants: 'lung cancer' OR 'lung carcinoma' OR 'lung tumor' OR 'lung tumour' OR 'lung neoplasm' OR 'lung malignancy' OR 'pulmonary cancer' OR 'pulmonary carcinoma' OR 'pulmonary tumor' OR 'pulmonary tumour' OR 'pulmonary neoplasm' OR 'pulmonary malignancy' OR 'NSCLC' OR 'non-small cell lung cancer' OR 'non small cell lung cancer' OR 'small cell lung cancer' OR 'SCLC'

Intervention: 'ALK inhibitor' OR 'anaplastic lymphoma kinase inhibitor' OR 'ALK TKI' OR 'ALK tyrosine kinase inhibitor' OR 'Alectinib' OR 'Alecensa' OR 'Lorlatinib' OR 'Lorbrena' OR 'Ensartinib' OR 'X-396' OR 'Iruplinalkib' OR 'WX-0593' OR 'Brigatinib' OR 'Alunbrig' OR 'Envonalkib' OR 'TQ-B3139'

Comparators: 'Crizotinib' OR 'Xalkori' OR 'PF-02341066'

Study Designs: 'randomized controlled trial' OR 'randomised controlled trial' OR 'RCT' OR random* OR 'random allocation' OR 'randomly assigned' OR compared OR comparison OR compare OR contrast OR versus OR equivalence OR switching OR equivalent OR comparing.

In addition, using similar search methodologies in Pubmed from inception until June 20, 2025., we have also identified case reports, pathogenic mechanisms, and contributing factors associated with ALK inhibitor-induced hepatotoxicity.

Appendix S2. The inclusion/exclusion criteria.

Category	Inclusion Criteria	Exclusion Criteria
Study Design	Randomized controlled trials	Non-randomized controlled trials studies (e.g. cohort study and case-control study)
Patients	Histologically/cytologically confirmed lung cancer (all types)	Non-lung cancer patients; mixed cancers
Intervention	ALK inhibitors	Other targeted agents
Comparison	Crizotinib	Not crizotinib (e.g. Placebo)
Outcomes	Hepatotoxicity incidence/severity	No hepatotoxicity data
Other	English publications; until June 2025	Non-English publications; Outside the specified time frame

Appendix S3. Statistical conversion of confidence intervals.

Since the Phase III trial of iruplinalkib reported the HR value for PFS with a 98.02% confidence interval, we converted its results to 95% CI to standardize the outcomes and facilitate pooled

analysis (. J Thorac Oncol. 2024 Jun;19(6):912-927.). To convert the reported hazard ratio (HR) of 0.34 with a 98.02% confidence interval (CI: 0.23–0.52) to a 95% CI, we assume the log-transformed HR follows a normal distribution. The conversion is performed by first calculating the Z-scores corresponding to both confidence levels: the original 98.02% CI uses a Z-score of 2.326 (derived from the standard normal distribution), while the target 95% CI uses 1.960. The standard error (SE) of the log (HR) is calculated as the width of the original log-scale CI divided by twice its Z-score:

$$SE = \frac{log\ (0.52) - log\ (0.23)}{2 \times 2.326} \approx 0.1754$$

Using this SE, the 95% CI on the log scale is computed. Exponentiation then provides the final 95% CI: HR=0.34 (95% CI:0.24–0.48). This narrower interval (0.24–0.48 vs. original 0.23–0.52) aligns with statistical theory, as reduced confidence precision contracts the bounds while maintaining the point estimate (0.34) near the center. The result suggests iruplinalkib significantly reduces progression risk relative to crizotinib, with 95% confidence the true HR lies between 0.24 and 0.48.

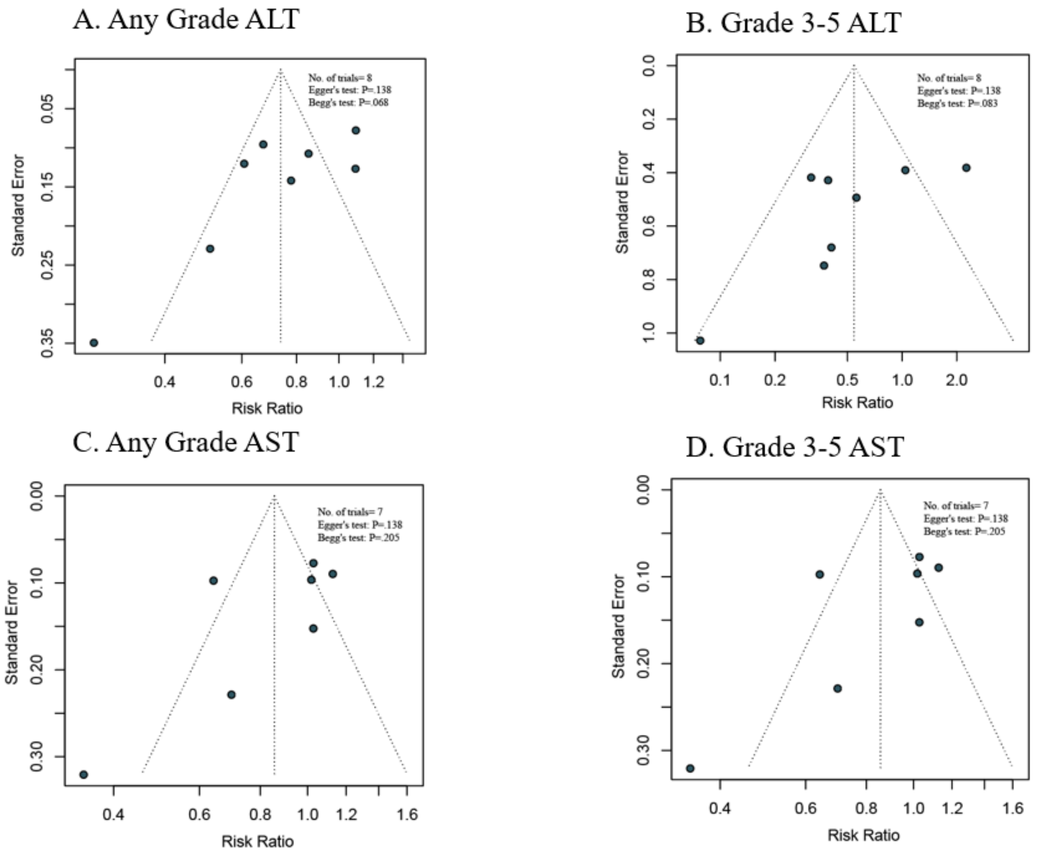

Figure S1. Evaluation of publications bias.

Abbreviations: ALT, alanine aminotransferase; AST, aspartate aminotransferase.

Table S1. Descriptions of hepatotoxicity in the approval labels for ALK inhibitors.

Gerneric name	Indications	Approval date		Warnings in the labeling		Dose change for the hepatic toxicities	
		FDA	NMPA	FDA	NMPA	FDA	NMPA
Crizotinib	For ALK-positive mNSCLC.	2011-08-26	2013-01-22	WARNINGS AND PRECAUTIONS section emphasizes that hepatotoxicity requires regular monitoring.	Similar to the FDA	1. ALT or AST >5×ULN with total bilirubin ≤1.5×ULN: withhold treatment until levels recover to baseline or ≤3×ULN, then resume at a reduced dose.<br>2.ALT or AST >3×ULN with total bilirubin >1.5×ULN (without cholestasis or hemolysis): permanently discontinue treatment.	Similar to the FDA
	For ROS1-positive advanced NSCLC.	2016-03-11	2017-10-17				
	For ALK-positive relapsed/refractory systemic ALCL in pediatric patients 1 year of age and older and young adults	2021-01-14	NA				
	For ALK-positive unresectable/recurrent/refractory IMT in pediatric patients 1 year of age and older and young adults	2022-07-14	NA				
Alectinib	For ALK+ mNSCLC.	2015-12-11	2018-08-12	WARNINGS AND PRECAUTIONS section emphasizes that hepatotoxicity requires regular monitoring.	Similar to the FDA	1.ALT or AST >5× ULN with total bilirubin ≤2× ULN: temporarily withhold treatment until levels recover to baseline or ≤3× ULN and Resume at a reduced dose;<br>2. ALT/AST >3× ULN + total bilirubin >2× ULN (without cholestasis/hemolysis): permanently discontinue.<br>3.Total bilirubin >3× ULN: withhold until recovery to baseline or ≤1.5× ULN, then resume at a reduced dose.	Similar to the FDA
	For adjuvant Treatment of ALK+ NSCLC.	2024-04-18	2024-06-28				

Gerneric name	Indications	Approval date		Warnings in the labeling		Dose change for the hepatic toxicities	
		FDA	NMPA	FDA	NMPA	FDA	NMPA
Lorlatinib	For ALK+ mNSCLC	2018-11-02	2022-04-27	WARNINGS AND PRECAUTIONS section emphasizes that hepatotoxicity requires regular monitoring.	Not highlighted	Not highlighted	Not highlighted
Brigatinib	For ALK+ mNSCLC	2017-04-28	2022-03-22	WARNINGS AND PRECAUTIONS section emphasizes that hepatotoxicity requires regular monitoring.	Not highlighted	1.ALT/AST >5× ULN (bilirubin ≤2× ULN): hold until recovery to Grade ≤1 (≤3× ULN) or baseline. Resume at next lower dose;<br>2. ALT/AST >3× ULN + bilirubin >2× ULN (no cholestasis/hemolysis): permanently discontinue.	Not highlighted
Ensartinib	For ALK+ mNSCLC	2024-12-18	2020-11-17	WARNINGS AND PRECAUTIONS section emphasizes that hepatotoxicity requires regular monitoring.	Similar to the FDA	1.ALT/AST >5× ULN (bilirubin ≤2× ULN): hold until recovery to Grade ≤1 (≤3× ULN) or baseline. Resume at next lower dose;<br>2. ALT/AST >3× ULN + bilirubin >2× ULN (no cholestasis/hemolysis): permanently discontinue.	Similar to the FDA
Iruplinalkib	For ALK+ mNSCLC	NA	2023-06-27	NA	WARNINGS AND PRECAUTIONS section emphasizes that hepatotoxicity requires regular monitoring.	NA	1.ALT/AST >5× ULN (bilirubin ≤2× ULN): hold until recovery to Grade ≤1 (≤3× ULN) or baseline. Resume at next lower dose;<br>2. ALT/AST >3× ULN + bilirubin >2× ULN (no cholestasis/hemolysis): permanently discontinue.

Gerneric name	Indications	Approval date		Warnings in the labeling		Dose change for the hepatic toxicities	
		FDA	NMPA	FDA	NMPA	FDA	NMPA
Envonalkib	For ALK+ mNSCLC	NA	2024-06-11	NA	WARNINGS AND PRECAUTIONS section emphasizes that hepatotoxicity requires regular monitoring.	NA	1.ALT/AST >5× ULN (bilirubin ≤2× ULN): hold until recovery to Grade ≤1 (≤3× ULN) or baseline. Resume at next lower dose;<br>2. ALT/AST >3× ULN + bilirubin >2× ULN (no cholestasis/hemolysis): permanently discontinue.
Ceritinib	For ALK+ mNSCLC	2014-04-29	2018-10-31	WARNINGS AND PRECAUTIONS section emphasizes that hepatotoxicity requires regular monitoring.	Similar to the FDA	1.ALT/AST >5× ULN (bilirubin ≤2× ULN): hold until recovery to Grade ≤1 (≤3× ULN) or baseline. Resume at next lower dose;<br>2. ALT/AST >3× ULN + bilirubin >2× ULN (no cholestasis/hemolysis): permanently discontinue.	Similar to the FDA

ALK, anaplastic lymphoma kinase; mNSCLC, metastatic non-small cell lung cancer; NMPA, National Medical Products Administration (China); FDA, Food and Drug Administration (US); ALT, alanine aminotransferase; AST, aspartate aminotransferase; ULN, upper limit of normal; BALCL, anaplastic large cell lymphoma; IMT, inflammatory myofibroblastic tumor; NA, not available.

Table S2. Summary of case reports on hepatotoxicity induced by ALK inhibitors.

Year	Author	Number of Cases	Age	Sex	Dosage	Duration of Exposure	Presenting Symptoms	Mortality	Treatment
**Crizotinib**									
2013	Ripault, et al[1].	1	69	F	500 mg QD	60 days	Fatigue	0	Withdrawal of medication with resolution
2014	Sato, et al[2].	1	54	F	400 mg QD	29 days	Epigastric pain	1	Withdrawal of medication, steroids, plasma exchange
2015	Tsukita, et al[3].	1	44	F	200 mg BID	15 days	Asymptomatic (lab abnormalities)	0	Dose reduction to alternate-day 250 mg BID
2016	Sassier, et al[4].	2	30, 62	M, F	250 mg QD	10 days, 30 days	None	0	Withdrawal of medication with resolution
2018	Adhikari, et al[5].	1	56	M	250 mg BID	39 days	Fatigue, nausea/vomiting	1	NAC, mannitol, withdrawal of medication, lactulose/rifaximin/LOLA/abx
2016	Van Geel, et al[6].	1	62	F	250 mg BID	24 days	Confusion	1	Supportive, withdrawal of medication
2017	Brown, et al[7].	1	56	F	250 mg BID	6 weeks	Fatigue, weakness, darkening urine	1	NAC, withdrawal of medication
2017	Yasuda, et al[8].	1	51	F	250 mg BID	16 days	Abdominal pain, nausea, diarrhea	1	Steroids, supportive, withdrawal of medication
2018	Charville, et al[9].	1	26	F	500 mg QD	10 weeks	Abdominal pain, jaundice	0	Withdrawal of medication with resolution
2019	Zhang, et al[10].	1	37	M	250 mg BID	55 days	Dyspnea, abdominal pain	1	Withdrawal of medication, lactulose/rifaximin/antibiotics
2019	Ota, et al[11].	1	66	F	250 mg BID	23 days	Fever, fatigue	0	Withdrawal of medication with resolution
2019	Kreitman, et al[12].	1	46	F	200 mg BID	8 weeks	Fatigue, abdominal pain, jaundice	0	Withdrawal of medication, NAC
2021	Duarte, et al[13].	1	32	F	250 mg BID	60 days	Nausea, fatigue	0	Withdrawal of medication, liver function recovery, switched to alectinib
2021	Yildiz, et al[14].	1	36	M	250 mg BID	4 months	Asymptomatic (lab abnormalities)	0	Withdrawal of medication, ursodeoxycholic acid, NAC, vitamin E
2023	Zhang, et al[15].	1	70	F	250 mg BID	9 weeks	Fatigue, anorexia, nausea, vomiting	0	Withdrawal of medication, liver protection drugs, corticosteroids
**Alectinib**									
2018	Zhu, et al[16].	1	42	F	600 mg BID	5 months	Asymptomatic (ALT/AST elevation)	0	Withdrawal, resolution after 4 weeks. Later rechallenged at same dose without recurrence.
2021	Peng, et al[17].	1	56	M	600 mg BID	119 days	Jaundice, pruritus, fatigue	0	Withdrawal, ALSS. Switched to ensartinib with no recurrence.
2021	Makimoto, et al[18].	1	57	M	600mg QD, 150 mg QD	79 days (initial), 64 days (rechallenge)	Cholestatic liver injury (AST/ALT elevation)	0	Withdrawal, UDCA/glycyrrhizin. Switched to lorlatinib with no hepatotoxicity.
2023	Juncu, et al[19].	1	73	M	600 mg BID	5 months	Jaundice, fatigue	0	Withdrawal, corticosteroids/NAC. Permanent discontinuation.

2024	Zhang, et al[20].	1	48	M	600 mg BID	3 years	Jaundice, abdominal distension	0	Withdrawal, ALSS. Liver biopsy confirmed DILI. Permanent discontinuation.
**Brigatinib**									
2023	Buyuksimsek, et al[21].	1	58	NR	NR	5 months	Liver enzyme elevation (>5×ULN)	0	Methylprednisolone with enzyme normalization
**Ceritinib**									
2018	Hirano, et al[22].	1	53	F	Not specified	1 month	Fever, elevated hepatobiliary enzymes	0	Discontinuation of certinib, prednisolone

ALT, alanine aminotransferase; AST, aspartate aminotransferase; ULN, upper limit of normal; QD, once daily; BID, twice daily; NAC, N-acetylcysteine; LOLA, L-ornithine L-aspartate; UDCA, ursodeoxycholic acid; ALSS, artificial liver support system; DILI, drug-induced liver injury; NSCLC, non-small cell lung cancer; NR, not reported.

Table S3. Search strategy.

Search strategy of PubMed		Numbers
#1	(Lung[Title/Abstract] OR pulmonary[Title/Abstract]) AND (cancer[Title/Abstract] OR carcinoma[Title/Abstract] OR tumor[Title/Abstract] OR tumour[Title/Abstract] OR neoplasm[Title/Abstract] OR malignant[Title/Abstract] OR oncology[Title/Abstract]) OR NSCLC[Title/Abstract] OR "non-small cell lung cancer"[Title/Abstract] OR "non small cell lung cancer"[Title/Abstract] OR SCLC[Title/Abstract] OR "small cell lung cancer"[Title/Abstract]	417728
#2	"ALK inhibitor"[Title/Abstract] OR "Anaplastic Lymphoma Kinase inhibitor"[Title/Abstract] OR "ALK TKI"[Title/Abstract] OR Alectinib[Title/Abstract] OR Lorlatinib[Title/Abstract] OR Ensartinib[Title/Abstract] OR Iruplinalkib[Title/Abstract] OR Brigatinib[Title/Abstract] OR Envonalkib[Title/Abstract]	2874
#3	Crizotinib[Title/Abstract] OR Xalkori[Title/Abstract] OR "PF-02341066"[Title/Abstract]	3514
#4	randomized[Title/Abstract] OR randomised[Title/Abstract] OR RCT[Title/Abstract] OR random*[Title/Abstract] OR "random allocation"[Title/Abstract] OR "randomly assigned"[Title/Abstract] OR compared[Title/Abstract] OR comparison[Title/Abstract] OR compare[Title/Abstract] OR versus[Title/Abstract] OR equivalence[Title/Abstract] OR equivalent[Title/Abstract]	7,703,934
#5	#1 AND #2 AND #3 AND #4	379
Search strategy of Embase		Numbers
#1	(lung:ab,ti OR pulmonary:ab,ti) AND (cancer:ab,ti OR carcinoma:ab,ti OR tumor:ab,ti OR tumour:ab,ti OR neoplasm:ab,ti OR malignant:ab,ti OR oncology:ab,ti) OR NSCLC:ab,ti OR 'non-small cell lung cancer':ab,ti OR 'non small cell lung cancer':ab,ti OR SCLC:ab,ti OR 'small cell lung cancer':ab,ti	650,227
#2	ALK inhibitor':ab,ti OR 'Anaplastic Lymphoma Kinase inhibitor':ab,ti OR 'ALK TKI':ab,ti OR Alectinib:ab,ti OR Lorlatinib:ab,ti OR Ensartinib:ab,ti OR Iruplinalkib:ab,ti OR Brigatinib:ab,ti OR Envonalkib:ab,ti	5,256
#3	Crizotinib:ab,ti OR Xalkori:ab,ti OR 'PF-02341066':ab,ti	6,933
#4	randomized:ab,ti OR randomised:ab,ti OR RCT:ab,ti OR random*:ab,ti OR 'random allocation':ab,ti OR 'randomly assigned':ab,ti OR compared:ab,ti OR comparison:ab,ti OR compare:ab,ti OR versus:ab,ti OR equivalence:ab,ti OR equivalent:ab,ti	10,744,897
#5	#1 and #2 and #3 and #4	915
Search strategy of Cochrane Library		Numbers
#1	(Lung OR pulmonary OR NSCLC OR "non-small cell lung cancer" OR "non-small cell lung cancer" OR SCLC OR "small cell lung cancer") AND (cancer OR carcinoma OR tumor OR tumour OR neoplasm OR malignant OR oncology):ti,ab,kw	40316

Search strategy of PubMed		Numbers
#2	("ALK inhibitor" OR "Anaplastic Lymphoma Kinase inhibitor" OR "ALK TKI" OR Alectinib OR Lorlatinib OR Ensartinib OR Iruplinalkib OR Brigatinib OR Envonalkib):ti,ab,kw	479
#3	(Crizotinib OR Xalkori OR "PF-02341066"):ti,ab,kw	482
#4	(randomized OR randomised OR RCT OR random* OR "random allocation" OR "randomly assigned" OR compared OR comparison OR compare OR versus OR equivalence OR equivalent):ti,ab,kw	1690445
#5	#1 and #2 and #3 and #4	275
Search strategy of ClinicalTrials.gov		Numbers
#1	(NSCLC OR "non-small cell lung cancer" OR SCLC OR "small cell lung cancer") AND ("ALK inhibitor" OR Alectinib OR Lorlatinib OR Ensartinib OR Iruplinalkib OR Brigatinib OR Envonalkib) AND Crizotinib AND (randomized OR randomised OR RCT OR random OR "random allocation" OR compared OR comparison OR versus) | Completed WITH RESULTS	6

Table S4. Mechanism and factors of ALK inhibitors-induced hepatotoxicity.

Year	Authors	ALK inhibitors	Study subjects	Mechanism/Factors
**Animal and cell research**				
2017	Zhang, et al[23]	Crizotinib	Isolated rat liver mitochondria	Crizotinib causes mitochondrial dysfunction only at supratherapeutic concentrations.
2018	Mingard, et al[24]	Crizotinib	HepG2 (glucose/galactose medium); HepaRG	Crizotinib causes apoptosis through non-mitochondrial pathways involving caspase-3 and PARP cleavage.
2019	Yan, et al[25]	Crizotinib	HL-7702; Human primary hepatocytes	Crizotinib induces ROS-mediated DNA damage and apoptosis independent of ALK/ROS1/MET targets.
2021	Guo, et al[26]	Crizotinib	Human L02 hepatocytes; C57BL/6J mice	Crizotinib promotes ROS accumulation and Nrf2 inhibition, triggering mitochondrial apoptosis.
2022	Li, et al[27]	Crizotinib	HL-7702 hepatocytes; C57BL/6J mice	Crizotinib triggers ROS-induced pyroptosis and autophagy via NF-κB/NLRP3 and mTOR pathways.
2022	Guo, et al[28]	Crizotinib	HepG2 cells; ICR mice	Crizotinib activates ROS-mediated mitochondrial apoptosis and Keap1/Nrf2 oxidative stress response.
2024	Wu, et al[29]	Ensartinib	C57BL/6J mice; Human primary hepatocytes; HL-7702; AML12; HEK293T; NCI-H3122	Ensartinib induces hepatotoxicity via TXNIP-mediated mitochondrial dysfunction and oxidative stress.
2024	Guo, et al[30]	Crizotinib	HL-7702; AML12; NCI-H2228 & H3122; C57BL/6J mice	Crizotinib induces ferroptosis by Stat1-mediated Nrf2 suppression and antioxidant depletion.
2025	Yan, et al[31]	Crizotinib	HL-7702; THLE-2; AML12; Human/mouse primary hepatocytes; C57BL/6J mice	Crizotinib blocks autophagic SQLE degradation, disturbing cholesterol/sphingolipid metabolism and apoptosis.
**Human study**				
2018	Jung, et al[32]	Crizotinib	NSCLC patients with ALK or ROS1 mutations	Crizotinib-induced hepatotoxicity is associated with pre-existing liver disease, HBV infection, and use of H2-antagonists or PPIs.
2019	Lin, et al[33]	Crizotinib	ALK/ROS1/MET-driven NSCLC	Sequential ICI/crizotinib treatment is associated with a significantly increased risk of hepatotoxicity
2021	Xin, et al[34]	Crizotinib	ALK+ NSCLC patients	STAT1 rs10208033 polymorphism is linked to higher risk of crizotinib-induced hepatotoxicity.
2022	Han, et al[35]	Crizotinib	TKI-treated patients	Male sex, liver metastasis, H2 blockers/PPIs, and anticancer drugs increase risk of TKI hepatotoxicity.

ALK, anaplastic lymphoma kinase; NSCLC, non-small cell lung cancer; PPIs, proton pump inhibitors; TKI, tyrosine kinase inhibitor.

Table S5. Clinical dose selection for ALK Inhibitors and dose optimization

Generic name	Phase I		Phase III		Conduct RCT for dose optimization	Approval dose in the labeling
	Trial number	MTD/MAD	Trial number	Dose		
Crizotinib	NCT00585195	MTD: 300 mg BID	NCT01154140	250 mg BID	No	250 mg BID
Alectinib	NCT04181060	MAD: 600 mg BID	NCT02838420	600 mg BID	No	600 mg BID
Lorlatinib	NCT03052608	MAD: 200 mg QD	NCT03052608	100 mg QD	No	100 mg QD
Brigatinib	NCT01449461	MAD: 300mg QD	NCT02737501	180 mg QD	Yes	180 mg QD
Ensartinib	NCT02959619	MTD: 225mg QD	NCT02767804	225 mg QD	No	225 mg QD
Iruplinalkib	NCT03389815	MTD: 300 mg QD	NCT04632758	180 mg QD	No	180 mg QD
Envonalkib	NCT03099330	MAD: 800 mg QD	NCT04009317	600 mg BID	No	600 mg BID

MTD, maximum tolerated dose; MAD, maximum administered dose; BID, twice daily; QD, once daily; RCT, randomized controlled trial; ALK, anaplastic lymphoma kinase.

Reference

1. Ripault, M.P., Pinzani, V., Fayolle, V., Pageaux, G.P., and Larrey, D. (2013). Crizotinib-induced acute hepatitis: first case with relapse after reintroduction with reduced dose. Clinics and research in hepatology and gastroenterology *37*, e21–23. 10.1016/j.clinre.2012.10.003.
2. Sato, Y., Fujimoto, D., Shibata, Y., Seo, R., Suginoshita, Y., Imai, Y., and Tomii, K. (2014). Fulminant hepatitis following crizotinib administration for ALK-positive non-small-cell lung carcinoma. Japanese journal of clinical oncology *44*, 872–875. 10.1093/jjco/hyu086.
3. Tsukita, Y., Fukuhara, T., Kobayashi, M., Morita, M., Suzuki, A., Watanabe, K., Noguchi, T., Kurata, Y., Suno, M., and Maemondo, M. (2015). Alternate-day Treatment with Crizotinib for Drug-induced Esophagitis and Liver Damage in a Patient with EML4-ALK Fusion Gene-positive Lung Adenocarcinoma. Internal medicine (Tokyo, Japan) *54*, 3185–3188. 10.2169/internalmedicine.54.4996.
4. Sassier, M., Mennecier, B., Gschwend, A., Rein, M., Coquerel, A., Humbert, X., Alexandre, J., Fedrizzi, S., and Gervais, R. (2016). Successful treatment with ceritinib after crizotinib induced hepatitis. Lung cancer (Amsterdam, Netherlands) *95*, 15–16. 10.1016/j.lungcan.2016.02.008.
5. Adhikari, N., Kumar, P., Venkatesulu, B.P., Pandey, R., Haresh, K.P., Gupta, S., Sharma, D.N., and Rath, G.K. (2018). Crizotinib-Induced Fulminant Hepatic Failure: A Rare Adverse Event. Journal of global oncology *4*, 1–4. 10.1200/jgo.2016.007765.
6. van Geel, R.M., Hendrikx, J.J., Vahl, J.E., van Leerdam, M.E., van den Broek, D., Huitema, A.D., Beijnen, J.H., Schellens, J.H., and Burgers, S.A. (2016). Crizotinib-induced fatal fulminant liver failure. Lung cancer (Amsterdam, Netherlands) *93*, 17–19. 10.1016/j.lungcan.2015.12.010.
7. Brown, P.R., Jones, D., Hassan, M., and Moonka, D. (2017). Crizotinib-Induced Fulminant Liver Failure: 2331. Official journal of the American College of Gastroenterology | ACG *112*, S1273.
8. Yasuda, Y., Nishikawa, Y., Sakamori, Y., Terao, M., Hashimoto, K., Funazo, T., Nomizo, T., Tsuji, T., Yoshida, H., Nagai, H., et al. (2017). Successful oral desensitization with crizotinib after crizotinib-induced hepatitis in an anaplastic lymphoma kinase-rearranged non-small-

cell lung cancer patient: A case report. Molecular and clinical oncology *7*, 295–297. 10.3892/mco.2017.1310.
9. Charville, G.W., Padda, S.K., Sibley, R.K., Puthillath, A., and Kwo, P.Y. (2018). Resolution of Crizotinib-Associated Fulminant Hepatitis following Cessation of Treatment. Case reports in hepatology *2018*, 3413592. 10.1155/2018/3413592.
10. Zhang, Y., Xu, Y.Y., Chen, Y., Li, J.N., and Wang, Y. (2019). Crizotinib-induced acute fatal liver failure in an Asian ALK-positive lung adenocarcinoma patient with liver metastasis: A case report. World journal of clinical cases *7*, 1080–1086. 10.12998/wjcc.v7.i9.1080.
11. Ota, T., Masuda, N., Matsui, K., Yamada, T., Tanaka, N., Fujimoto, S., and Fukuoka, M. (2019). Successful Desensitization with Crizotinib after Crizotinib-induced Liver Injury in ROS1-rearranged Lung Adenocarcinoma. Internal medicine (Tokyo, Japan) *58*, 2651–2655. 10.2169/internalmedicine.2554-18.
12. Kreitman, K., Nair, S.P., and Kothadia, J.P. (2020). Successful Treatment of Crizotinib-Induced Fulminant Liver Failure: A Case Report and Review of Literature. Case reports in hepatology *2020*, 8247960. 10.1155/2020/8247960.
13. Duarte, F.A., Rodrigues, L.B., Paes, F.R., Diniz, P.H.C., and Lima, H. (2021). Successful treatment with alectinib after crizotinib-induced hepatitis in ALK-rearranged advanced lung cancer patient: a case report. BMC pulmonary medicine *21*, 43. 10.1186/s12890-020-01390-6.
14. Yildiz, I. (2021). Liver and Pancreatic Injury in Response to ALK Inhibitors in a Patient with Primary Signet Ring Cell Carcinoma of the Lung: A Case Report. Case reports in oncology *14*, 107–111. 10.1159/000512829.
15. Zhang, X., Ni, K., and Chen, H. (2023). Successful Retreatment with Crizotinib After Crizotinib-Induced Liver Failure in ALK-Positive Advanced Lung Adenocarcinoma: A Case Report. OncoTargets and therapy *16*, 87–90. 10.2147/ott.s393165.
16. Zhu, V.W., Lu, Y., and Ou, S.I. (2019). Severe Acute Hepatitis in a Patient Receiving Alectinib for ALK-Positive Non-Small-Cell Lung Cancer: Histologic Analysis. Clinical lung cancer *20*, e77–e80. 10.1016/j.cllc.2018.09.006.
17. Peng, L., Xiao, K., Cui, J., Ye, X.H., Zhang, Y.C., Mao, L., Selvaggi, G., Yen, J., and Stebbing, J. (2021). Successful Treatment with Ensartinib After Alectinib-induced Hyperbilirubinemia in ALK-Positive NSCLC. OncoTargets and therapy *14*, 3409–3415. 10.2147/ott.s310756.
18. Makimoto, G., Kawakado, K., Nakanishi, M., Tamura, T., and Kuyama, S. (2021). Successful Treatment with Lorlatinib after the Development of Alectinib-Induced Liver Damage in ALK-Positive Non-Small-Cell Lung Cancer: A Case Report. Case reports in oncology *14*, 197–201. 10.1159/000513624.
19. Juncu, S., Trifan, A.V., Minea, H., Avram, R.I., Cojocariu, C., and Sîngeap, A.M. (2023). From spotlight to shadow: ALK inhibitor-induced acute liver failure in a patient with non-small cell lung cancer. Archive of clinical cases *10*, 160–163. 10.22551/2023.41.1004.10266.
20. Zhang, Q., Yan, L., Bao, Y., Yuan, X., Yin, D., and Xu, J. (2024). Hyperbilirubinemia in a Patient Receiving Alectinib for Anaplastic Lymphoma Kinase Positive Non-Small-Cell Lung Cancer: A Histological Features. OncoTargets and therapy *17*, 1189–1193. 10.2147/ott.s486860.
21. Buyuksimsek, M., Ogul, A., Yetisir, A.E., Duman, B.B., Tohumcuoglu, M., Cil, T., Koseci,

T., Kidi, M.M., and Sumbul, H.E. (2023). Brigatinib-associated autoimmune hepatitis: A case report. Journal of oncology pharmacy practice : official publication of the International Society of Oncology Pharmacy Practitioners, 10781552231171322. 10.1177/10781552231171322.

22. Hirano, T., Koarai, A., Ichikawa, T., Sato, T., Ohe, T., and Ichinose, M. (2018). Possible involvement of interleukin-18 in the pathology of hepatobiliary adverse effects related to treatment with ceritinib. BMC cancer *18*, 995. 10.1186/s12885-018-4913-5.
23. Zhang, J., Salminen, A., Yang, X., Luo, Y., Wu, Q., White, M., Greenhaw, J., Ren, L., Bryant, M., Salminen, W., et al. (2017). Effects of 31 FDA approved small-molecule kinase inhibitors on isolated rat liver mitochondria. Archives of toxicology *91*, 2921–2938. 10.1007/s00204-016-1918-1.
24. Mingard, C., Paech, F., Bouitbir, J., and Krähenbühl, S. (2018). Mechanisms of toxicity associated with six tyrosine kinase inhibitors in human hepatocyte cell lines. Journal of applied toxicology : JAT *38*, 418–431. 10.1002/jat.3551.
25. Yan, H., Du, J., Chen, X., Yang, B., He, Q., Yang, X., and Luo, P. (2019). ROS-dependent DNA damage contributes to crizotinib-induced hepatotoxicity via the apoptotic pathway. Toxicology and applied pharmacology *383*, 114768. 10.1016/j.taap.2019.114768.
26. Guo, L., Gong, H., Tang, T.L., Zhang, B.K., Zhang, L.Y., and Yan, M. (2021). Crizotinib and Sunitinib Induce Hepatotoxicity and Mitochondrial Apoptosis in L02 Cells via ROS and Nrf2 Signaling Pathway. Frontiers in pharmacology *12*, 620934. 10.3389/fphar.2021.620934.
27. Li, M., Wang, C., Yu, Z., Lan, Q., Xu, S., Ye, Z., Li, R., Ying, L., Zhang, X., and Zhou, Z. (2022). MgIG exerts therapeutic effects on crizotinib-induced hepatotoxicity by limiting ROS-mediated autophagy and pyroptosis. Journal of cellular and molecular medicine *26*, 4492–4505. 10.1111/jcmm.17474.
28. Guo, L., Tang, T., Fang, D., Gong, H., Zhang, B., Zhou, Y., Zhang, L., and Yan, M. (2022). An Insight on the Pathways Involved in Crizotinib and Sunitinib Induced Hepatotoxicity in HepG2 Cells and Animal Model. Frontiers in oncology *12*, 749954. 10.3389/fonc.2022.749954.
29. Wu, W., Li, J., Yin, Y., Zhou, Y., Huang, X., Cao, Y., Chen, X., Zhou, Y., Du, J., Xu, Z., et al. (2024). Rutin attenuates ensartinib-induced hepatotoxicity by non-transcriptional regulation of TXNIP. Cell biology and toxicology *40*, 38. 10.1007/s10565-024-09883-4.
30. Guo, L., Ma, J., Xiao, M., Liu, J., Hu, Z., Xia, S., Li, N., Yang, Y., Gong, H., Xi, Y., et al. (2024). The involvement of the Stat1/Nrf2 pathway in exacerbating Crizotinib-induced liver injury: implications for ferroptosis. Cell death & disease *15*, 600. 10.1038/s41419-024-06993-z.
31. Yan, H., Huang, X., Zhou, Y., Mu, Y., Zhang, S., Cao, Y., Wu, W., Xu, Z., Chen, X., Zhang, X., et al. (2025). Disturbing Cholesterol/Sphingolipid Metabolism by Squalene Epoxidase Arises Crizotinib Hepatotoxicity. Advanced science (Weinheim, Baden-Wurttemberg, Germany) *12*, e2414923. 10.1002/advs.202414923.
32. Jung, D., Han, J.M., Yee, J., Kim, J.Y., and Gwak, H.S. (2018). Factors affecting crizotinib-induced hepatotoxicity in non-small cell lung cancer patients. Medical oncology (Northwood, London, England) *35*, 154. 10.1007/s12032-018-1213-5.
33. Lin, J.J., Chin, E., Yeap, B.Y., Ferris, L.A., Kamesan, V., Lennes, I.T., Sequist, L.V., Heist,

R.S., Mino-Kenudson, M., Gainor, J.F., and Shaw, A.T. (2019). Increased Hepatotoxicity Associated with Sequential Immune Checkpoint Inhibitor and Crizotinib Therapy in Patients with Non-Small Cell Lung Cancer. Journal of thoracic oncology : official publication of the International Association for the Study of Lung Cancer *14*, 135–140. 10.1016/j.jtho.2018.09.001.

34. Xin, S., Fang, W., Li, J., Li, D., Wang, C., Huang, Q., Huang, M., Zhuang, W., Wang, X., and Chen, L. (2021). Impact of STAT1 polymorphisms on crizotinib-induced hepatotoxicity in ALK-positive non-small cell lung cancer patients. Journal of cancer research and clinical oncology *147*, 725–737. 10.1007/s00432-020-03476-4.

35. Han, J.M., Yee, J., Cho, S., Kim, M.K., Moon, J.Y., Jung, D., Kim, J.S., and Gwak, H.S. (2022). A Risk Scoring System Utilizing Machine Learning Methods for Hepatotoxicity Prediction One Year After the Initiation of Tyrosine Kinase Inhibitors. Frontiers in oncology *12*, 790343. 10.3389/fonc.2022.790343.
